# Management of otitis externa with an led-illuminated gel: a randomized controlled clinical trial in dogs

**DOI:** 10.1186/s12917-020-02311-9

**Published:** 2020-03-20

**Authors:** Adolfo Maria Tambella, Anna Rita Attili, Francesca Beribè, Margherita Galosi, Andrea Marchegiani, Matteo Cerquetella, Angela Palumbo Piccionello, Cecilia Vullo, Andrea Spaterna, Alessandro Fruganti

**Affiliations:** School of Biosciences and Veterinary Medicine, University of Camerino, Via Circonvallazione, 93/95, 62024 Matelica, MC Italy

**Keywords:** Otitis, Otitis externa, Photobiomodulation, Fluorescence biomodulation, Biophotonics, Phototherapy, Light, Topical administration, Dog, Inflammation

## Abstract

**Background:**

Canine otitis externa is a painful condition which can be challenging to treat due to difficulties in the administration of otic medication. This can be due to lack of owner compliance in the application of ear drops or due to the resentment that some dogs demonstrate when attempts are made to administer topical medication into a sensitive ear canal. The aim of the study was to assess the efficacy of a topical LED-illuminated gel (*LIG*) in canine otitis externa in comparison to standard of care therapy. Dogs with spontaneous otitis externa were randomly allocated in three groups: groups QW received *LIG* once weekly; BW received *LIG* twice weekly; group C received enrofloxacin and silver sulfadiazine twice daily. *LIG* consists of a topical application of a gel containing chromophores that, when illuminated by a LED lamp, re-emit fluorescent light which can stimulate physiological responses, promoting healing and controlling bacteria. The evaluation protocol (T_0_ to T_5_) considered clinical assessment (OTIS-3-index-scoring-system; pruritus-severity-scale; pain-severity-score; aural temperature), cytological scoring system, quali-quantitative bacteriologic assessment.

**Results:**

All groups (QW, *n* = 21; BW, *n* = 23; C, *n* = 20) showed improvement during the study (QW: *P* < 0.02 for cytological and pain scores, *P* < 0.003 for bacteriologic assessment, *P* < 10^− 4^ for pruritus, total OTIS-3 and temperature assessments; BW: *P* < 10^− 4^ for all clinical, cytological and bacteriologic assessments; C: *P* < 0.02 for all clinical and cytological assessments, *P* < 10^− 4^ for bacteriologic assessment). The highest clinical score reduction occurred in Group BW (*P* < 0.014 in T_3_; *P* < 0.001 in T_4_ and *P* < 10^− 4^ in T_5_). BW reached the clinically relevant effect level at T_3_ (− 3.26 ± 1.21 levels), QW reached it at T_4_ (− 3.24 ± 0.99), C did not reach it. No differences between groups were seen in the reduction of CFU/mL (T_0_-T_5_).

**Conclusions:**

All treatment groups showed a positive clinical effect. *LIG* administered twice-a-week was the most favourable protocol of the study. *LIG* may be considered beneficial in the management of canine otitis externa; it seems to be effective in controlling the clinical condition, including the signs of inflammation and local pain, the bacterial growth, and it may help increasing treatment compliance.

## Background

Several studies showed that otitis externa is one of the most frequent diagnosis in small animal practice [1–3]. Many different factors are involved in the aetiopathogenesis of otitis externa. Primary causes which underlie all cases of canine otitis include ectoparasites, allergy, keratinization disorders and autoimmune disease. Infection is always defined as a secondary cause and will not occur in a normal ear. Predisposing factors include conformational factors, excessive moisture, iatrogenic factors and obstructive ear disease. Perpetuating factors which drive the otitis once it has been established and need to be treated to prevent recurrence are those caused through chronic disease such as a chronic inflammatory state and the progressive pathologic changes within the canal and otitis media [4–11].

Otitis externa has been reported to greatly decrease the quality of life of both dogs and owners [12, 13]. Dogs with otitis have disturbed sleep patterns, interact less with their owners and will often resent being handled due to the pain of the otitis. Owners complain that administering therapy to dogs with otitis is time consuming, often unpleasant due to the presence of a malodorous discharge and many have difficulty treating large, strong or refractory dogs [12, 13]. The net result is a deterioration in the dog-owner relationship and poor owner compliance leading to possible treatment failure due to courses of treatment not being completed. Where otitis recurs due to short comings in treatment application the inflammation and infection will inevitably become more challenging to treat and changes within the ear will become more chronic, on occasions progressing to irreversible damage which can only be treated surgically [14, 15]. Although ablative surgery can help bring comfort to these chronic patients, it is considered a highly invasive and painful procedure [14], not free from intraoperative and postoperative complications [16].

One study by Noli [17] considered the benefits in terms of clinical efficacy, owner compliance and quality of life for owner and pet of a long-acting gel containing an antibiotic-antimycotic-glucocorticoid combination, administered by the veterinary surgeon once weekly. Conclusions from the work suggested that when a veterinarian administered an otic gel it provided equivalent efficacy but a higher quality of life to dogs with otitis externa and their owners, compared to an owner administering a topical otic therapy [17]. Based on the above-noted study, it would appear that owners favour products, in this case an antibiotic, that do not have to be used at home and prefer medication that is administered on a less frequent basis by their veterinarian. However, such a conclusion does not address the global drive towards responsible antibiotic stewardship and the real need to develop products that can accelerate healing to reduce or avoid the use of antibiotics.

It would seem therefore that a product that can be applied by the veterinarian on a weekly or biweekly basis that can control the acute and developing chronic disease state, while being able to control bacteria should have a place in the therapeutic armoury for otitis externa.

In this context, photobiomodulation (PBM) is widely known for its therapeutic benefits in the protection and regeneration of tissues [18–23]. Studies have demonstrated that PBM can reduce pain and inflammation [23–25], improve cancer management [26], and stimulate healing and tissue repair [20–22, 27–31]. PBM is defined by the use of visible light to stimulate biological functions in a non-thermal and non-cytotoxic manner. Advances in understanding how PBM achieves its biological impact have identified endogenous photoacceptors that are widely expressed in different cells types, including skin cells, as well as in the extracellular matrix. Interactions between light and these photoacceptors have been demonstrated to modulate biological processes, including inflammation, the control of bacteria, angiogenesis, and signal transduction pathways that recruit transcription factors activating several genes involved in multiple aspects of cell biology [32].

Fluorescence biomodulation (FB), a form of PBM that uniquely employs fluorescence light energy (FLE), has been demonstrated to advance healing of both acute and chronic wounds [22, 29–31, 33]. A study has demonstrated that acute incisional wounds have reduced inflammation, as well as more physiologic re-epithelization and collagen remodelling, resulting in better quality and less visible scars [21]. In a multicenter, observational, uncontrolled trial, patients with hard-to-heal chronic ulcers experienced accelerated healing and improved quality of life [22].

The LED-illuminated gel (*LIG*) consists of two components: a light source comprised of blue light emitting diodes (LEDs; peak wavelength between 440 and 460 nm) and a topical substrate containing chromophores. These FB substrates are constructs, generally of silicone- or nylon-based membranes or amorphous hydrogels, optimized for different therapeutic uses and delivery of photonic energy. Of note, the substrates themselves are not absorbed by the tissue [30, 34]; their impact is achieved through the light energy delivered to the tissue.

In vitro studies evaluated the potential mechanisms of action behind FB technology and how it modulates cellular activity in inflammatory dermatological conditions. FB using *LIG* showed high capacity to enhance collagen production in human dermal fibroblasts; attenuate the inflammatory reaction by significantly reducing the release of tumor necrosis factor alpha (TNF-α) and interleukin-6 (IL-6) from both human dermal fibroblast and human embryonic kidney cells; enhance angiogenesis in human aortic endothelial cells increasing both microvascular tube and branching points formation, similarly to vascular endothelial growth factor (VEGF) a potent angiogenic factor [35]. Furthermore, in biopsies from canine chronic deep pyoderma treated with *LIG*, an increase in the number and size of mitochondria occurred, demonstrating an increase in mitochondrial activity [36].

Recent studies have shown that *LIG* has beneficial effect on wound healing in dogs [37]; an excellent safety profile and efficacy have also been shown in canine pyoderma [38] and otitis [39].

The purpose of the study was to determine whether topical *LIG* can be beneficial in the management of canine otitis externa. The hypothesis was that *LIG* compared with standard of care (SOC) therapy, shows positive effect on otitis externa in dogs.

## Results

Sixty-four otitic ears from 37 dogs of 18 different breeds were included in the study (QW, *n* = 21; BW, *n* = 23; C, *n* = 20) (Table 1). Mixed breed dogs (36.0%), German shepherd dogs (12.5%), English bulldogs (6.2%), Italian hound (6.2%), Springer spaniels (4.7%) were the most commonly represented breeds.

**Table 1:** Comparison of baseline demographic and clinical data in the three groups on day 0

	Group QW	Group BW	Group C	Statistical data
	(*n* = 21)	(*n* = 23)	(*n* = 20)	***P***-value
**Age** (mean ± sd; months)	100.0 ± 31.4	106.4 ± 45.7	91.8 ± 45.6	F = 0.66 *P* = 0.520
**Body weight** (mean ± sd; Kg)	23.1 ± 11.2	23.4 ± 11.6	18.9 ± 10.4	F = 1.06 *P* = 0.352
**Gender** (F:M ratio; number of cases)	9:12	13:10	11:9	χ^2^ = 0.958 *P* = 0.619
**Exudate type** (C:S ratio; number of cases)	12:9	7:16	6:14	χ^2^ = 4.293 *P* = 0.117

*QW* group receiving *LIG* once weekly, *BW* group receiving *LIG* twice weekly, *C* group receiving *standard of care* twice daily, *sd* standard deviation, *F* female, *M* male, *C* ceruminous exudate, *S* suppurative exudate

The final sample size (*n* = 64) was greater than the minimum required sample size (*n* = 57) calculated a priori and required for a reliable RCT (effect size 0.4327121; alpha-error 0.05; actual power 0.8191020). At final *post-hoc* analysis, the achieved power of the study was 99.36% (total sample size 64; effect size 0.6147541, alfa-error 0.05).

No technical problems occurred during the topical application. The exogenous chromophores in the topical gel responded to the LED light illumination, with a visible colour change of the gel from orange to pink occurring in all treatment applications. The application was well tolerated without apparent side or adverse events. All patients completed the application and evaluation protocol.

### Clinical assessments

#### Otitis index scoring system (OTIS-3)

**Fig. 1:**
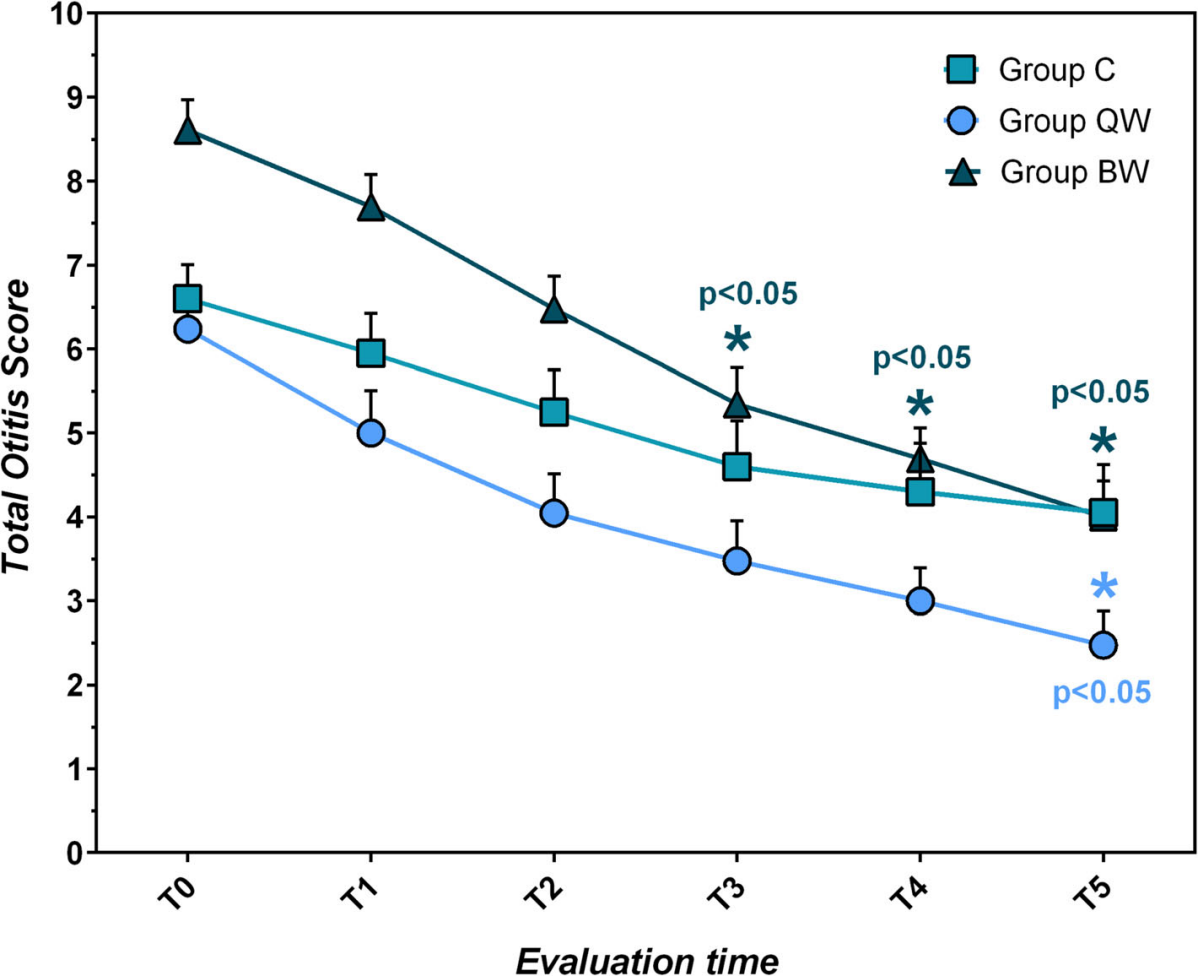
Trend of mean Total OTIS-3 Index Scoring System (total score, 0 to 12) ± SEM in the study groups during the trial, with indication (*) of the statistical significance (*p* < 0.05), considering the mean OTIS-3 reduction, between Group BW and Group C, and between Group QW and Group C

All three groups showed lowering of clinical score (OTIS-3) during the whole trial (C: χ^2^_r_ = 63.313, *P* < 10^− 4^; QW: χ^2^_r_ = 81.214, *P* < 10^− 4^; BW: χ^2^_r_ = 98.937, *P* < 10^− 4^) with significant effect appreciated in all groups already at T_2_ (C: q = 4.243, *P* < 0.05; QW: q = 5.016, *P* < 0.05; BW: q = 4.848, *P* < 0.05) and lasted for the whole follow up (Table 2; Fig. 1).

**Table 2:** Comparison of Otitis Index Scoring System (OTIS-3) within each group from T_0_ to T_5_

	T_**0**_	T_**5**_	Statistical data;
	(mean score ± sd)	(mean score ± sd)	***P***-value
**Group C**	6.60 ± 1.82	4.05 ± 2.56	W = 190; *P* < 0.02
**Group QW**	6.24 ± 2.09	2.48 ± 1.83	W = 231; *P* < 10^−4^
**Group BW**	8.61 ± 1.72	4.00 ± 2.04	W = 276; *P* < 10^−4^

*QW* group receiving *LIG* once weekly, *BW* group receiving *LIG* twice weekly, *C* group receiving standard of care twice daily, *sd* standard deviation, *T*_*0*_ first evaluation time, *T*_*5*_ sixth (last) evaluation time

Considering the mean OTIS-3 reduction, significant differences between groups were observed at T_3_ (H = 8.505, *P* < 0.014), at T_4_ (H = 13.679, *P* < 0.001) and at T_5_ (H = 17.053, *P* < 10^− 4^). The comparison BW vs C showed significant differences at T_3_ (BW: − 3.26 ± 1.21; C:-2.00 ± 1.41; Q = 2.914, *P* < 0.05), at T_4_ (BW: − 3.91 ± 1.59; C: − 2.30 ± 1.52; Q = 3.698; *P* < 0.05), and at T_5_ (BW: − 4.61 ± 1.53; C: − 2.55 ± 1.68; Q = 4.117, *P* < 0.05). The comparison QW vs C showed significant difference at T_5_ (QW: − 3.76 ± 1.22; C: − 2.55 ± 1.64; Q = 2.433, *P* < 0.05). No significant differences in clinical OTIS-3 were found comparing Groups QW and BW (Fig. 1).

In all groups the maximum lowering of the clinical score was reached at T_5_ (BW: − 4.61 ± 1.53 levels; QW: − 3.76 ± 1.22 levels; C: − 2.55 ± 1.64 levels). Group BW reached the clinically relevant effect at T_3_ (− 3.26 ± 1.21 levels), QW reached it at T_4_ (− 3.24 ± 0.99), C did not reach the cut-off.

#### Pruritus severity scale

**Fig. 2:**
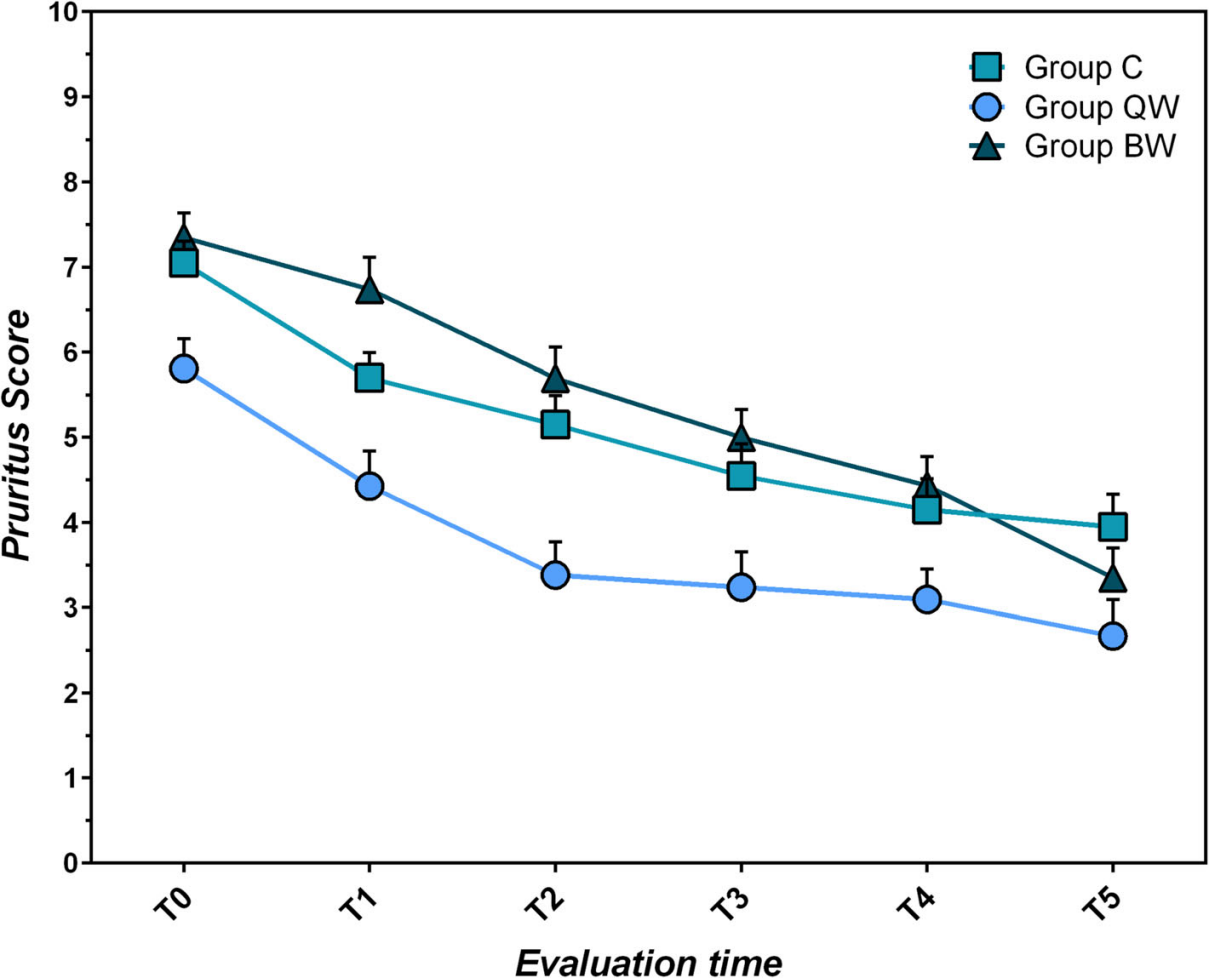
Mean Pruritus Severity Scores (VAS, 0 to 10) ± SEM per study group

All three groups showed lowering of Pruritus Severity Scale during the trial (C: χ^2^_r_ = 53.148, *P* < 10^− 4^; QW: χ^2^_r_ = 48.978, *P* < 10^− 4^; BW: χ^2^_r_ = 91.291, *P* < 10^− 4^) (Table 3; Fig. 2). No significant differences between groups were found for pruritus severity score (*P* > 0.05).

**Table 3:** Comparison of pruritus severity scale within each group from T_0_ to T_5_

	T_**0**_	T_**5**_	Statistical data;
	(mean score ± sd)	(mean score ± sd)	***P***-value
**Group C**	7.05 ± 1.14	3.95 ± 1.70	W = 190; *P* < 0.02
**Group QW**	5.81 ± 1.16	2.67 ± 1.96	W = 219; *P* < 10^−4^
**Group BW**	7.35 ± 1.40	3.35 ± 1.69	W = 276; *P* < 10^− 4^

*QW* group receiving *LIG* once weekly, *BW* group receiving *LIG* twice weekly, *C* group receiving standard of care twice daily, *sd* standard deviation, *T*_*0*_ first evaluation time, *T*_*5*_ sixth (last) evaluation time

#### Pain severity score

**Fig. 3:**
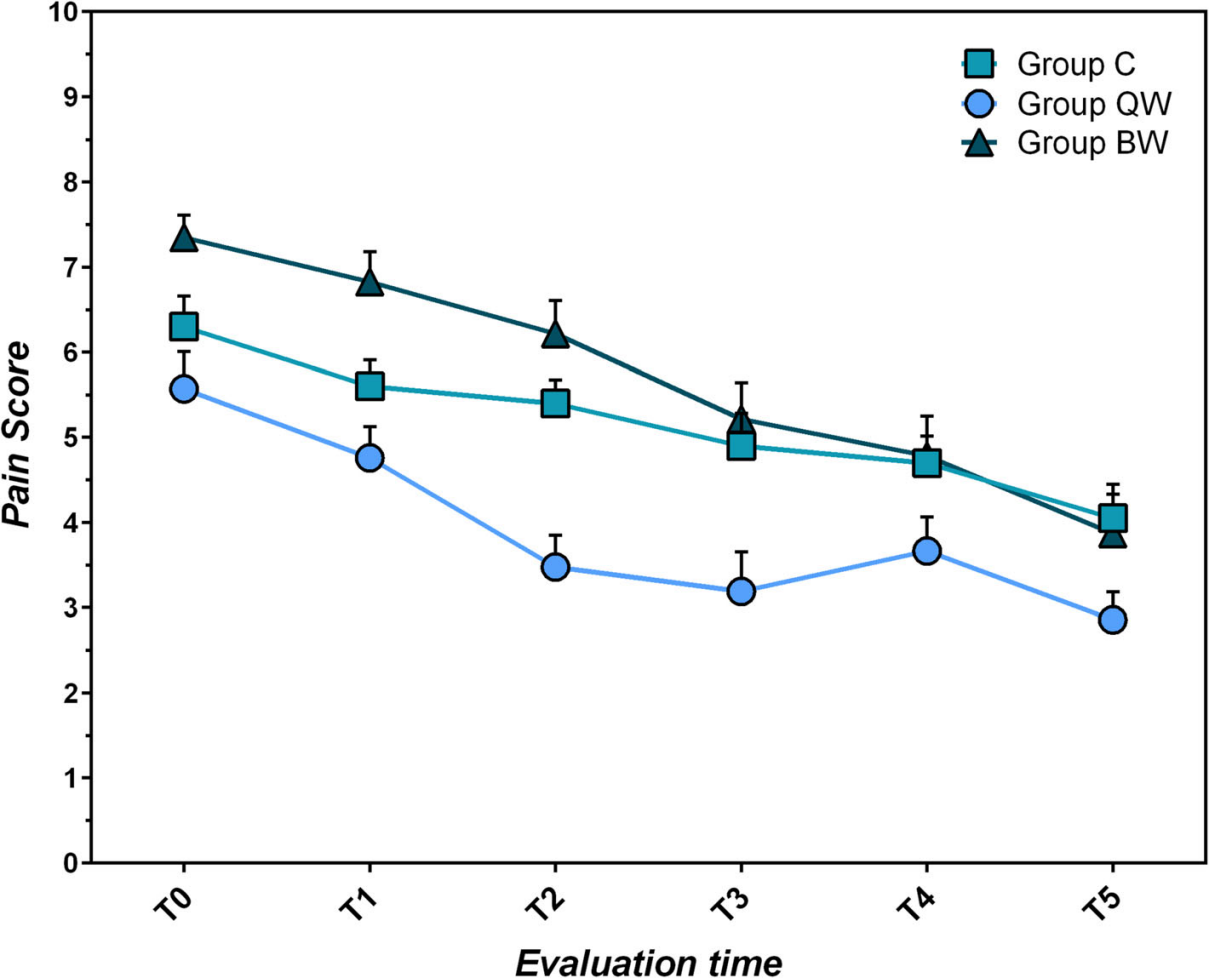
Mean Pain Severity Scores (VAS, 0 to 10) ± SEM per study group

All three groups showed lowering of Pain Severity Score during the trial (C: χ^2^_r_ = 39.053, *P* < 10^− 4^; QW: χ^2^_r_ = 37.614, *P* < 10^− 4^; BW: χ^2^_r_ = 83.000, *P* < 10^− 4^) (Table 4; Fig. 3). No significant differences between groups were found for pain severity score (*P* > 0.05).

**Table 4:** Comparison of pain severity score within each group from T_0_ to T_5_

	T_**0**_	T_**5**_	Statistical data;
	(mean score ± sd)	(mean score ± sd)	***P***-value
**Group C**	6.3 ± 1.59	4.05 ± 1.79	W = 159; *P* < 0.02
**Group QW**	5.57 ± 1.99	2.86 ± 1.49	W = 195; *P* < 0.02
**Group BW**	7.35 ± 1.26	3.87 ± 2.22	W = 253; *P* < 10^−4^

*QW* group receiving *LIG* once weekly, *BW* group receiving *LIG* twice weekly, *C* group receiving standard of care twice daily, *sd* standard deviation, *T*_*0*_ first evaluation time, *T*_*5*_ sixth (last) evaluation time

#### Aural temperature measurement

**Fig. 4:**
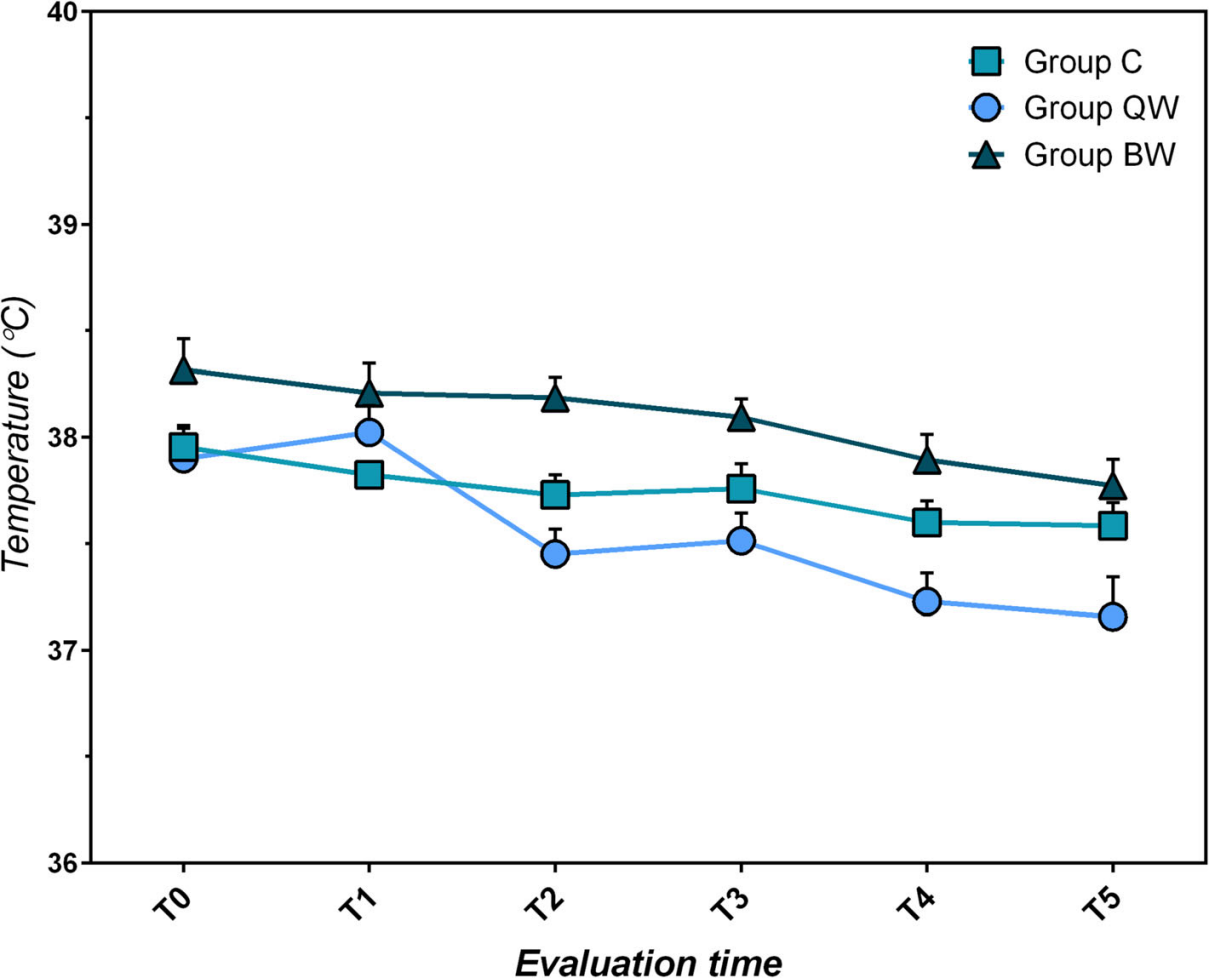
Mean Aural Temperatures (°C) ± SEM measured before application per study group

All three groups showed lowering of aural temperature (measured before treatment) during the trial (C: F = 3.833, *P* = 0.003; QW: F = 13.857, *P* < 10^− 4^; BW: F = 8.127, *P* < 10^− 4^) (Table 5; Fig. 4). No significant differences between groups were found considering aural temperature (*P* > 0.05).

**Table 5:** Comparison of aural temperature (°C) within each group from T_0_ to T_5_

	T_**0**_	T_**5**_	Statistical data;
	(mean score ± sd)	(mean score ± sd)	***P***-value
**Group C**	37.96 ± 0.39	37.58 ± 0.49	t = 3.225; *P* = 0.004
**Group QW**	37.90 ± 0.72	37.16 ± 0.85	t = 4.865; *P* < 10^−4^
**Group BW**	38.32 ± 0.70	37.77 ± 0.59	t = 4.976; *P* < 10^−4^

*QW* group receiving *LIG* once weekly, *BW* group receiving *LIG* twice weekly, *C* group receiving standard of care twice daily, *sd* standard deviation, *T*_*0*_ first evaluation time, *T*_*5*_ sixth (last) evaluation time

**Fig. 5:**
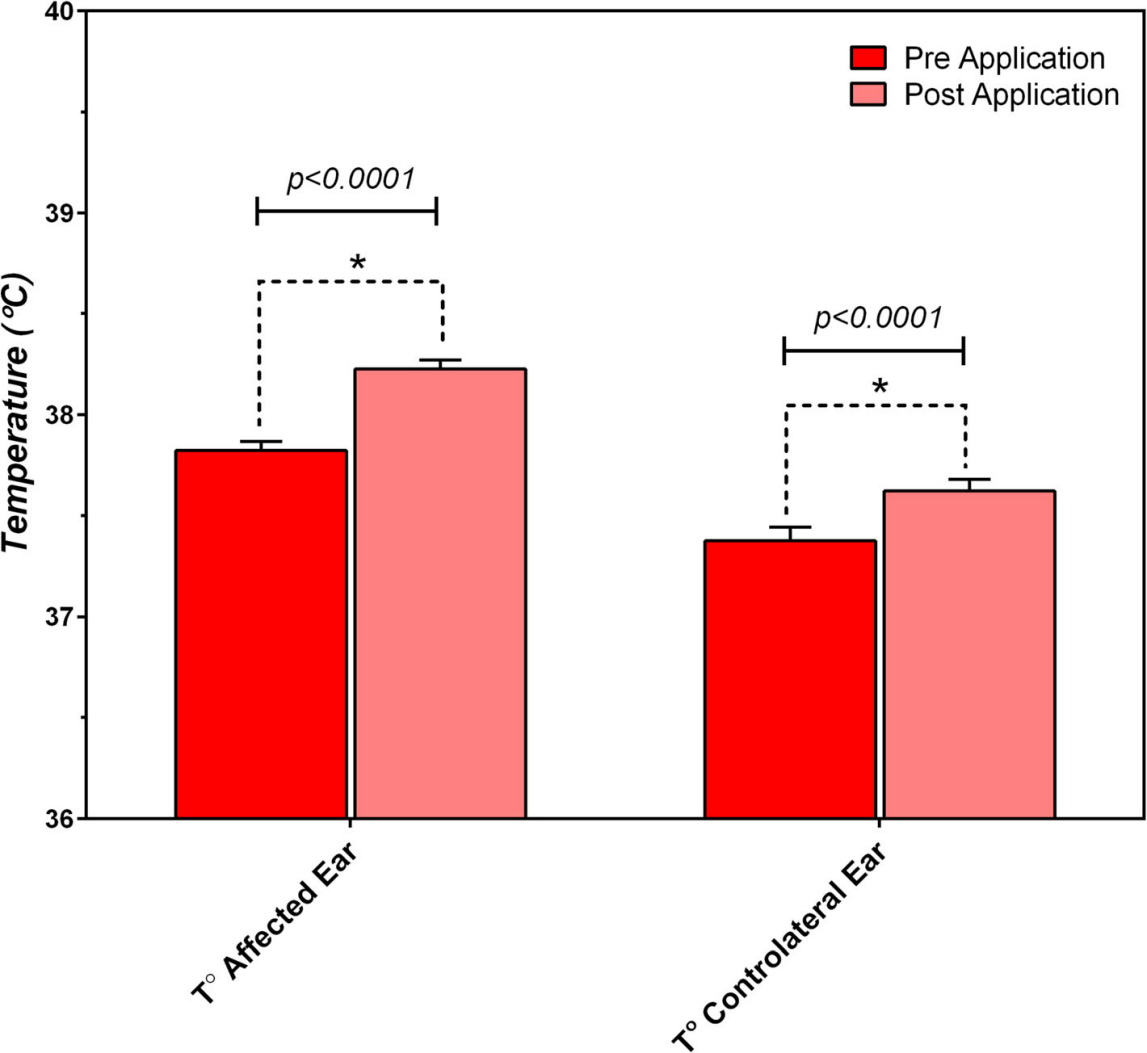
Mean Aural Temperature ± SEM measured before and immediately after application in exposed ear canals and in contralateral ear canals (°C)

When comparing aural temperature measured before and immediately after application in the whole *LIG* population (Groups QW and BW), both affected ear canals (in unilateral and bilateral otitis) and contralateral ear canals (in unilateral otitis) showed a significantly greater variation of aural temperature (+ 0.40 ± 0.37 °C, t = 17.5165, *P* < 0.0001 and + 0.24 ± 0.18 °C, t = 8.7780, *P* < 0.0001 respectively). In unilaterally affected dogs, a significant difference between affected ears and contralateral ears also occurred (t = 2.679, *P* = 0.0078). (Fig. 5) Despite the moderate increase in the aural temperature, no dog clinically manifested signs of discomfort during application of *LIG*. All ear canals, both affected and contralateral, returned to each baseline level of aural temperature within 5 min of treatment.

### Cytological assessment

**Fig. 6:**
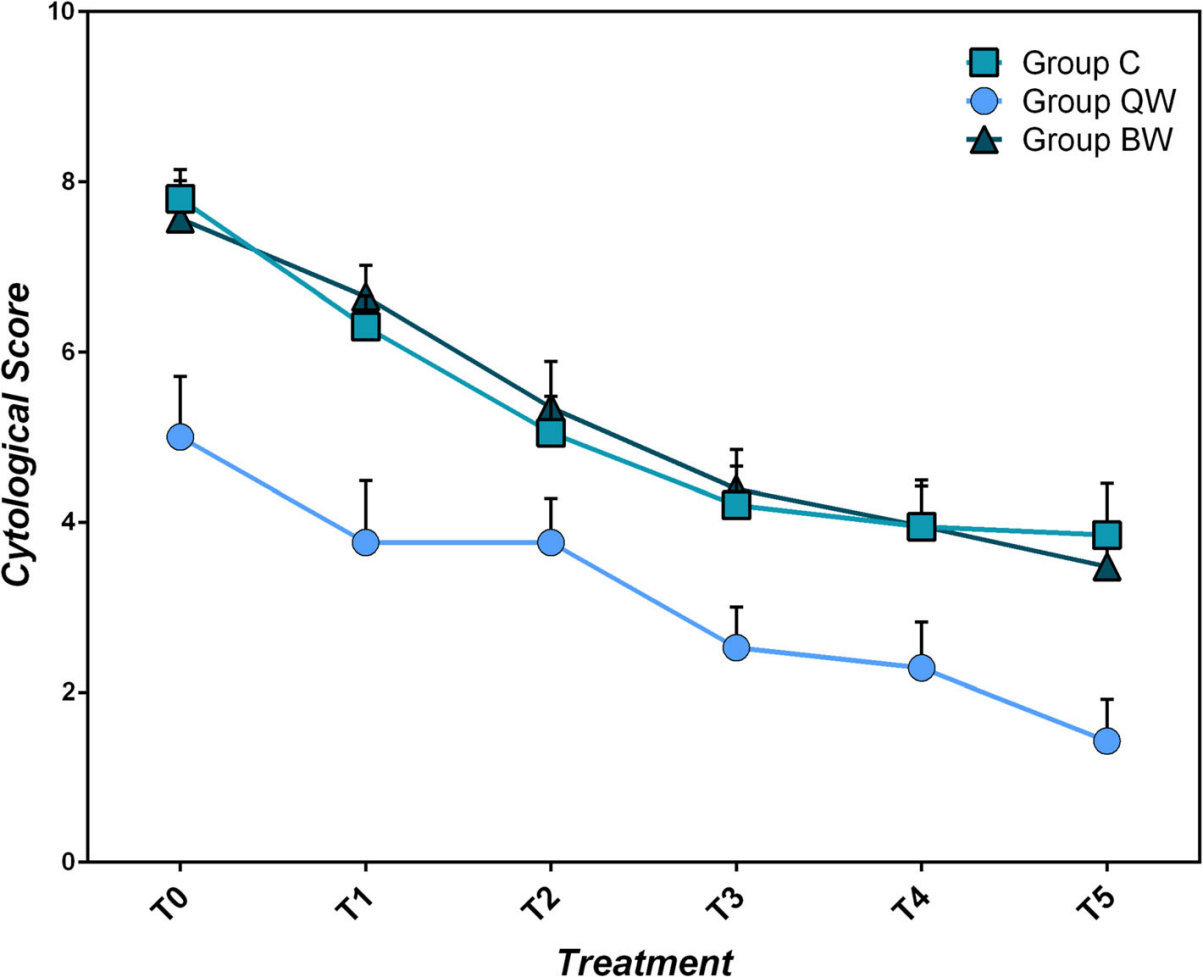
Mean Cytological Scoring System (total score, 0 to 12) ± SEM by study group

All three groups showed lowering of Otitis Cytological Scoring System during the trial (C: χ^2^_r_ = 67.476, *P* < 10^− 4^; QW: χ^2^_r_ = 57.476, *P* < 10^− 4^; BW: χ^2^_r_ = 59.327, *P* < 10^− 4^), with significant effect appreciated at T_1_ in Group C (q = 2.928, *P* < 0.05), at T_2_ in Group BW (q = 3.789, *P* < 0.05) and at T_4_ in Group QW (q = 5.949, *P* < 0.05). (Table 6; Fig. 6). No significant differences between groups were found for the cytological assessment (*P* > 0.05).

**Table 6:** Comparison of cytological assessment within each group from T_0_ to T_5_

	T_**0**_	T_**5**_	Statistical data;
	(mean score ± sd)	(mean score ± sd)	***P***-value
**Group C**	7.80 ± 1.54	3.85 ± 2.71	W = 210; *P* < 0.02
**Group QW**	5.00 ± 3.27	1.43 ± 2.25	W = 187; *P* < 0.02
**Group BW**	7.56 ± 2.15	3.48 ± 2.09	W = 253; *P* < 10^−4^

*QW* group receiving *LIG* once weekly, *BW* group receiving *LIG* twice weekly, *C* group receiving standard of care twice daily, *sd* standard deviation, *T*_*0*_ first evaluation time, *T*_*5*_ sixth (last) evaluation time

### Assessment of bacterial levels

**Fig. 7:**
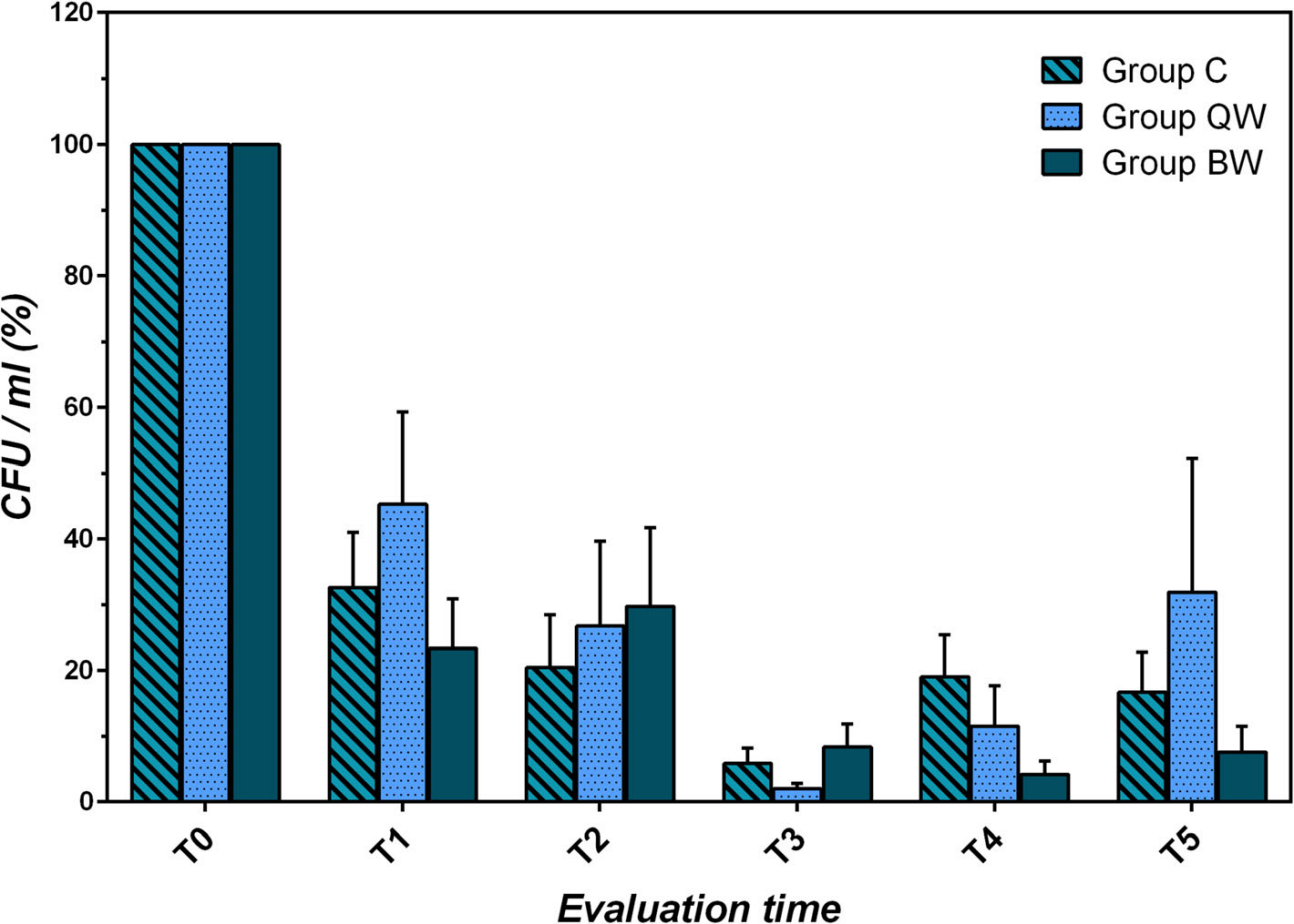
Trend of mean percentage of CFU/mL in relation to initial bacterial count (T_0_, 100%) in the three study groups during the trial

All three groups showed a lowering of bacterial count during the trial. (Fig. 7) Significant reductions in CFU/mL were observed within all groups (Group C: *t* = 13.588, *P* < 10^− 4^; Group QW: *t* = 3.346, *P* = 0.003; Group BW: *t* = 23.428, *P* < 10^− 4^).

No significant differences were found at each observational time between groups considering bacterial CFU/mL (*P* > 0.05). At the end of the study, Group BW (− 92.39 ± 18.91) showed the highest, but not significant (F = 1.02; *P* = 0.367), mean percentage of reduction of CFU/mL (T_0_-T_5_), in comparison to Groups QW (− 68.10 ± 93.26) and C (− 83.29 ± 27.41) (Fig. 7).

Comparing the total bacterial count (CFU/mL) from swabs taken before and immediately after each *gel* application (Groups QW and BW), during the trial, a total number of 227 reductions (mean percentage of variation: − 93.13 ± 17.60%, median − 99.96) were observed, of which 73 became negative (− 100.00%); and a total number of 6 increases (mean percentage of variation: + 24,714.13 ± 59,258.57%, median + 681.82) were observed.

Considering the bacterial Gram staining, an overall decrease of bacterial frequency of isolation was observed within each group in both Gram-positive (*P* < 0.0005) and Gram-negative (*P* < 0.01) bacteria. In the Groups QW (χ^2^ = 11.250; *P* = 0.0008) and C (χ^2^ = 6.759; *P* = 0.0093), a greater decrease of the frequency isolation was observed in Gram-negative bacteria. In Group BW, both Gram-staining frequencies of isolation decreased with no statistical difference between Gram-positive and Gram-negative (χ^2^ = 0.696; *P* = 0.4042).

**Fig. 8:**
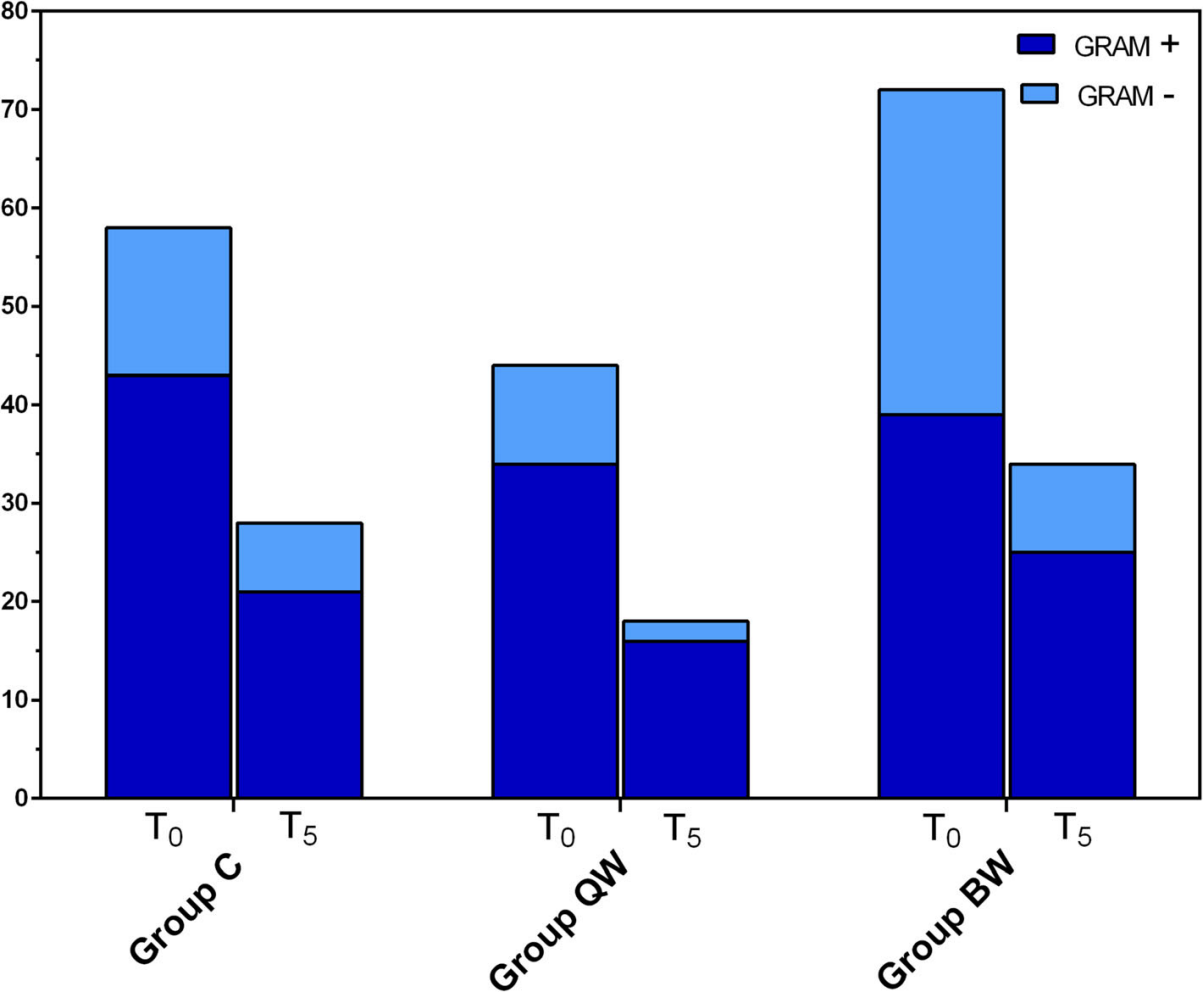
Frequency of bacterial isolation according to the Gram staining in Groups C, QW and BW, at the beginning (T_0_) and at the end (T_5_) of the trial

No significant statistical difference was observed between groups both considering Gram-positive (χ^2^ = 0.796; *P* = 0.672) and Gram-negative (χ^2^ = 1.250; *P* = 0.535) bacteria (Fig. 8).

## Discussion

The aim of this study was to assess the potential benefits of topical administration of *LIG* in the management of canine otitis externa.

The aetiopathogenesis of otitis externa is multifactorial and extremely varied so it may be particularly difficult to perform a reliable evaluation procedure during a clinical trial. In this study we opted for a broad evaluation protocol to take into consideration various clinical, cytological and bacteriological assessments. The authors consider that, for this complex pathology, there is no single assessment that can be considered sufficient on its own and hence a diversified evaluation protocol was applied, in order to best understand the potential benefits of this new therapeutic approach in the management of otitis. The OTIS-3 scoring scale was used as a primary outcome because it takes into consideration several parameters closely linked to the clinical aetiology of the disease. The parameters, namely erythema, oedema/swelling, erosion/ulceration and exudate are clinically relevant and additionally reflect the signs of the immune and inflammatory responses [40, 41] that would be taking place as the disease subsides. A change in the scoring would thus be indicative of clinical resolution.

In line with the reporting guidelines for RCTs [42], the appropriate sample size for the study was a-priori calculated to determine the number of cases required to detect a clinically relevant difference between the trial groups in the primary outcome. The minimum sample size was exceeded and a *post-hoc* power calculation confirmed the study was adequately powered to detect a difference between the groups. Sample size and power calculation is actually considered a crucial factor in the reliability of RCT results although it has been shown to be still vastly underreported in the veterinary trials literature [43–45].

The study showed that all three management protocols were effective clinically, cytologically and microbiologically. *LIG* groups showed better results in the primary outcome evaluation, especially Group BW, indicating that this particular management schedule can have an equivalent effect to the standard of care used, if not superior. As well, cytological assessment in *LIG* groups showed similar trend to SOC. The lack of significant statistical differences between groups in the clinical secondary outcomes (Pruritus Severity Scale; Pain Severity Score; Aural temperature) helps demonstrate that *LIG* can have at least an equivalent clinical effect to the standard of care chosen. These results are very encouraging and well in line with the previous results reported with FB systems in humans suffering from diseases of the skin, capable of accelerating healing, with reduced pain, and minimizing the clinical signs of bacterial colonization [22, 29, 32, 46–50].

Poly-microbial colonization often occurs in otitis externa and the continuum between mild–moderate colonization and the beginning of infection are blurred [51–53]. Bacteriological assessment in this study showed that the use of *LIG* indeed favoured improvement of clinical condition with a low rate of bacterial growth, as evidenced by the results presented in this study, in line with other previous trials [22].

Finally, the temporary, moderate increase in the temperature of the ear canal measured before and after application has not been clinically relevant, since no dog showed signs of discomfort. It also occurred in both affected and contralateral ear canals, so it was not only due to the heating induced by the lamp but probably also due to stress during manipulations.

Daily application of topical ear formulations in dogs at home is often difficult to perform leading to stress for both pets and owners and often poor treatment compliance [10, 17, 54]. A recent study has shown that reducing the frequency of otitis treatment can improve pets and owners quality of life [17]. The *LIG* system is designed to be applied in-clinic by a veterinarian or a trained veterinary nurse/assistant, relieving the owners from therapy application and ensuring that the product is always correctly applied, and at the right frequency. This could help achieve higher levels of owner compliance an improve the quality of life in both the dog and owner [17]. For those cases where home application of topicals is near impossible, *LIG* should also present a very appealing alternative.

The effect demonstrated by *LIG* in this study warrants further investigations. Of particular interest could be the synergistic effect when used concurrently with an antibiotic or potentially as an alternative to current standard management therapies to improve the overall clinical outcome of individual patients.

### Limitations

One of the limitations of the study is the difference in OTIS-3 scores between groups at enrolment, with the BW group averaging a higher condition severity than QW and C. This is also observed in some of the secondary parameters measured, although baseline demographic and clinical data did not show significant statistical differences. This limitation is intrinsic to the fact that it is a clinical trial with patients suffering from spontaneous pathology. To limit the weakness of the study potentially resulting from this factor and other sources of bias, a randomized allocation to the study group was performed; furthermore, the evaluation protocol considered, whenever possible, the execution of evaluations using variables that have been normalized to the initial condition of each group, thus considering, in many cases, relative variations and not the absolute variations.

Cytological and microbiological assessments were performed blindly. However, it was not possible to maintain blindness for clinical outcome assessments, since after the application of *LIG* it was difficult to completely remove the coloured gel, leaving a pale pink coloration of the skin and hairs. In many instances it was therefore possible to identify the cases that underwent topical application of *LIG* compared to cases treated with SOC.

Another limitation was that, to be included in this trial, dogs had to have an intact tympanic membrane visible by otoscopy before treatment. This selection criterion, proposed as a precaution to limit any potential risk of ototoxicity induced by the application of *LIG* or standard of care treatment, eliminated a number of cases with very hyperplastic ear canals, in which visualization of the tympanic membrane was prevented.

## Conclusions

An overall effect was found during the study both for *LIG* and for standard of care. The *LIG* administered twice weekly was the protocol that showed the greatest overall reduction in OTIS-3 score from start to finish of therapy course.

The *LED-Illuminated Gel* (*LIG*) may be considered beneficial in the management of canine otitis externa; it seems to be effective in improving clinical condition, modulating inflammation and controlling bacteria. Having demonstrated similar efficacy to SOC, it may help reducing or avoiding antibiotic treatment in otitis cases.

The lower frequency of application of *LIG* compared to standard of care could also increase therapeutic compliance.

## Methods

### Inclusion criteria

Dogs of different breed, age and sex, with unilateral or bilateral otitis externa were included in the study. A general physical examination was undertaken of all dogs including an otoscopic examination of the external ear canal. Dogs showing at least two signs consistent with otitis externa (erythema, pruritus, pain, swelling, ulceration, ceruminous or purulent discharge) were, with informed owner consent, enrolled in the study.

### Exclusion criteria

Animals whose owners had not signed the informed consent; animals with non-intact tympanic membrane; animals who had received during the last 2 weeks, topically and/or systemically, anti-inflammatory and/or antibiotic therapy; animals receiving photosensitizing molecules were excluded from the study.

### Study design

Three-arms prospective randomized controlled clinical trial (RCT).

### Application protocol

The protocol was reviewed and accepted by the Ethical Committee for the Protection of Animals of University of Camerino (Prot. 1–10.02.2017).

Included cases were randomly divided in three groups, considering the single ear as unit of analysis. The random assignment to groups was performed using the random number generator GraphPad QuickCalcs Software.

Group QW received topical application of *LIG* once weekly for six times; group BW received topical application of *LIG* twice weekly for six times; group C received standard of care topical therapy twice daily for 3 weeks.

*LIG* application consisted of the following steps, performed at each session both in group BW and QW: the chromophore gel was introduced into the external auditory canal until this was filled till the ear canal entrance (the volume of the chromophore gel introduced inside the ear canal was 1.5–4 mL depending on the size of the ear canal); a gentle massage of the ear canal was performed to facilitate uniform distribution of the gel throughout the external ear canal; the gel was photoactivated by inserting the tip of the LED lamp (Bluephase Lamp), covered by a single-use plastic sleeve, into the external auditory canal that was illuminated for 1.5 min, using soft start program (light intensity from 650 to 1200 mW/cm^2^) for 30 s and high power program (light intensity fixed at 1200 mW/cm^2^) for 1 minute; following the application, a generous rinsing of the external auditory canal with sterile saline solution was performed and dry gauze facilitated the removal of any gel residues.

Conventional topical therapy for otitis externa (performed in group C) consisted of topical administration of Baytril Otic® inside the external auditory canal (5–10 and 10–15 drops in dog weighing up to 16 kg and more than 16 kg, respectively). Baytril Otic® is an emulsion containing enrofloxacin (5 mg/mL) and silver sulfadiazine (10 mg/mL) with benzyl alcohol (20 mg/mL), cetyl stearyl alcohol in a neutral oil and purified water emulsion.

No concomitant anti-inflammatory and antibiotic, topical or systemic, therapies were carried out during the study, other than Baytril Otic® in Group C.

### Evaluation protocol and outcome measures

The evaluation protocol was made during treatment days for a total of six times in each group (T_0_ to T_5_), twice weekly for BW and C groups (with T_5_ reached in 3 weeks) and once weekly for QW group (with T_5_ reached in 6 weeks). It consisted of clinical assessments (Otitis Index Scoring System; Pruritus Severity Scale; Pain Severity Score; Aural temperature), cytological scoring system; quali-quantitative bacteriologic assessment.

After completing the study, the animals were released to the owner’s care.

#### Clinical assessments

The *Otitis Index Scoring System (OTIS-3)*, as described by Nuttal and Bensignor in 2014 [40], was used to assess the degree of severity of otitis at the beginning and during the protocol. In full accordance to the OTIS-3 protocol, four clinical parameters were scored: erythema, oedema/swelling, erosion/ulceration, exudate. For each clinical parameter, the scores ranked from 0 (none) to 3 (the most severe score). In each case the total OTIS-3 score could range from 0 to 12. OTIS-3 was considered the primary outcome of the trial. For the purpose of this study, a “clinically relevant effect” was determined as a 3-point reduction in OTIS-3 clinical score.

*Pruritus Severity Scale* was used to assess the clinical manifestation of spontaneous pruritus due to otitis, as described by Hill and Coll. in 2007 [55], Rybnicek and Coll. in 2008 [56], Hill and Coll. in 2010 [57]. The Pruritus Severity Scale ranged in a visual analogic scale (VAS), from 0 (none) to 10 (the most severe pruritus score).

*Pain Severity Score* was used to assess the clinical manifestation of pain induced by manipulation during evaluation and treatment procedures, as described by Buback and Coll. in 1996 [58], Wolfe and Coll. in 2006 [14], Nuttal and Bensignor in 2014 [40]. The Pain Severity Score ranged in a visual analogic scale (VAS), from 0 (none) to 10 (the most severe pain score).

*Aural temperature* was measured in Celsius degree (°C) before and after treatment, in the exposed and the contralateral ear canal (in case of unilateral otitis), as described by Grono in 1970 [59], Cole in 2009 [60], Mittal in 2014 [61].

#### Cytological assessment

An ear swab for cytological assessment was taken by the investigator during each evaluation time, for a total of six times (T_0_ to T_5_). The investigator assigned a secret identification code to each cytology slide so a blinded cytological assessment could be performed. The pathologist was blinded to the study group, treatment and study time. An Otitis Cytological Scoring System was used to assess semi-quantitatively the presence and quantity of five items: neutrophils, earwax/cerumen, rod shaped bacteria, coccoid bacteria, fungi/yeasts. For each items listed above the scores ranked from 0 (none) to 3 (high amount). In each case the total cytological score could range from 0 to 15. Each ear swab specimen was examined under low magnification (× 100) to find an area of interest and after that under × 400 magnification to count cells and microorganisms. Ten selected fields were counted and the mean number of cells and organisms per high-powered field calculated. For neutrophils, a score of 0 was assigned where no neutrophils were seen, 1 for < 10, 2 for 11–20 and 3 for > 20. For coccoid bacteria, a score of 0 was assigned for 0–3, 1 for 4–6, 2 for 7–25 and 3 for > 25. For rod shaped bacteria, a score of 0 was assigned where no rod bacteria were seen, 1 for < 6, 2 for 6–25 and 3 for > 25. For fungi/yeasts, a score of 0 was assigned for ≤2, 1 for 3–4, 2 for 5–8 and 3 for > 8. For earwax/cerumen a score of 0 was assigned where cerumen was absent, 1 for few presence, 2 for moderate presence, 3 for high presence.

#### Assessment of bacterial levels

During each evaluation time, before and after each treatment, ear swabs were collected for bacterial investigations. A total of 12 ear swabs in six times (T_0_ to T_5_) were sampled. The investigator assigned a secret identification code to each one so a blinded assessment could be performed. Moreover, the microbiologist of the Laboratory of Medical Microbiology and Infectious Diseases was blinded to the study groups, treatments, study time and time of collection. To assess the bacterial levels, mean of the total bacteria loads (CFU, colony forming units), and bacterial cultures, were carried out.

To assess the total bacterial count, each swab was immersed for 5 min in 1 mL of sterile 0.9% saline solution (Oxoid, Milan, Italy). Subsequently the sample was vortexed for 30 s and 100 μL were sown in duplicate by spatulating on the surface of Columbia Agar plates containing 5% sheep blood and incubated in an aerobic atmosphere for 24–48 h. On each plate the number of colony-forming units (CFU) was converted into number of bacteria colonies per 1 mL of solution (equal to the number of bacteria present in the swab using the equation of Ferguson and Coll, 2003) [62].

For qualitative bacteriological assessment, bacterial cultures were performed. Each swab underwent a pre-enrichment using Tryptic Soy Broth (Liofilchem, Italy) and incubated at 37 °C for 6-h. Then, each sample was spread onto Columbia Agar plate containing 5% sheep blood, with and without Streptococcus supplement, Mannitol Salt agar, Mac Conkey agar, Pseudomonas Cetrimide agar and *Burkholderia cepacia* selective agar (Liofilchem, Italy). Plates were incubated at 37 °C for 24–72 h in aerobic conditions. Gram positive bacteria were identified by Gram staining, catalase and coagulase tests, colony morphology and using commercial biochemical gallery (Remel RapID, ThermoFisher, Milan, Italy). Gram negative bacteria were identified by Gram staining, oxidase testing and commercial biochemical gallery (Remel Rapid ID, ThermoFisher, Milan, Italy).

### Statistical analysis

The minimum sample size was calculated a-priori using ANOVA method (power at least 80%; alpha-error 0.05) and effect size obtained from a preliminary report of the study [39]. At the end of the study, a *post-hoc* power analysis was performed on final sample size and effect size. Sample size and power analyses were performed with G-Power software, version 3.1.9.2.

Categorical variables were analysed and compared between groups using Qui-squared test. McNemar test was used to compare paired categorical variables within each group.

Ordinal variables were analysed with Kruskal-Wallis test followed by Dunn post-hoc test to obtain a comparison between the three groups. Friedman test, followed by Student-Newmann-Keuls post-hoc test was used to perform a comparison by study time within each group. A comparison T_0_*vs*T_5_ was performed within each group using Wilcoxon signed rank test.

Cardinal variables were analysed with One-way ANOVA followed by Holm-Sidak post-hoc test, to perform a comparison between groups. ANOVA for repeated measures, followed by Holm-Sidak post-hoc test, was used to compare by study time within groups. A comparison T_0_*vs*T_5_ was performed within each group using Paired Student-t-test.

A difference with a *P*-value ≤0.05 was considered statistically significant.

All data were statistically analysed with Primer of Biostatistics software, version 6.0.

## Data Availability

All relevant data is contained within the manuscript. The datasets used during this study are available from the corresponding author on request.

## Abbreviations

°C: Celsius degree
ANOVA: Analysis of variance
CFU: Colony-forming unit
EDTA: Ethylene Diamine Triacetic Acid
FB: Fluorescence biomodulation
FLE: Fluorescence light energy
LED: Light-emitting diode
LIG: LED-illuminated gel
mg/mL: Milligrams per milliliter
mW/cm^2^: Milliwatts per square centimeter
OTIS-3: Otitis Index Scoring System
PBM: Photobiomodulation
RCT: Randomised controlled clinical trial
SEM: Standard error of the mean
SOC: Standard of care
VAS: Visual analogic scale

## References

[1] Scott DW, Miller WH, Griffin CE (2001). Muller & Kirk’s small animal dermatology.

[2] Hill PB, Lo A, Eden CA, Huntley S, Morey V, Ramsey S (2006). Survey of the prevalence and treatment of dermatological conditions in small animals in general practice. Vet Rec.

[3] O’Neill DG, Church DB, McGreevy PD, Thomson PC, Brodbelt DC (2014). Prevalence of disorders recorded in dogs attending primary-care veterinary practices in England. PLoS One.

[4] Jacobson LS (2002). Diagnosis and medical treatment of otitis externa in the dog and cat. J S Afr Vet Assoc.

[5] Rosser EJ (2004). Causes of otitis externa. Vet Clin North Am Small Anim Pract.

[6] Saridomichelakis MN, Farmaki R, Leontides LS, Koutinas AF (2007). Aetiology of canine otitis externa: a retrospective study of 100 cases. Vet Dermatol.

[7] Craig M (2008). Disease facts: Otitis externa. UK Vet Companion Animal.

[8] Coatesworth J (2011). Causes of otitis externa in the dog. UK Vet Companion Animal.

[9] Zur G, Lifshitz B, Bdolah-Abram T (2011). The association between the signalement, common causes of canine otitis externa and pathogens. J Small Anim Pract.

[10] Forster SL, Real T, Doucette KP, King SB (2018). A randomized placebo-controlled trial of the efficacy and safety of a terbinafine, florfenicol and betamethasone topical ear formulation in dogs for the treatment of bacterial and/or fungal otitis externa. BMC Vet Res.

[11] Paterson S, Matyskiewick W (2018). A study to evaluate the primary causes associated with Pseudomonas otitis in 60 dogs. J Small Anim Pract.

[12] Noli C, Colombo S, Cornegliani L (2011). Quality of life of dogs with skin disease and of their owners. Part 2: administration of a questionnaire in various skin diseases and correlation to efficacy of therapy. Vet Dermatol.

[13] Jamet JF, Marignac G, Petit JY, Woehrle F, Petit JL, Perrot S (2016). Prospective study of the effect of otitis externa before and after treatment on 20 owners’ assessment of their own and their dog’s quality of life. Vet Dermatol.

[14] Wolfe TM, Bateman SW, Cole LK, Smeak DD (2006). Evaluation of al local anesthetic delivery system for the postoperative analgesic management of canine total ear canal ablation – a randomized, controlled, double-blinded study. Vet Anaesth Analg.

[15] Bacon NJ, Tobias KM, Johnston SA (2012). Pinna and external ear canal. Veterinary surgery: small animal.

[16] Coleman KA, Smeak DD (2016). Complication rates after bilateral versus unilateral total ear canal ablation with lateral bulla osteotomy for end-stage inflammatory ear disease in dogs: 79 ears. Vet Surg.

[17] Noli C, Sartori R, Cena T (2017). Impact of a terbinafine–florfenicol–betamethasone acetate otic gel on the quality of life of dogs with acute otitis externa and their owners. Vet Dermatol.

[18] Avci P, Gupta A, Sadasivam M, Vecchio D, Pam Z, Pam N (2013). Low-level laser (light) therapy (LLLT) in skin: stimulating, healing, restoring. Semin Cutan Med Surg.

[19] Hamblin MR (2016). Photobiomodulation or low-level laser therapy. J Biophotonics.

[20] Kulkarni S, Meer M, George R (2019). Efficacy of photobiomodulation on accelerating bone healing after tooth extraction: a systematic review. Lasers Med Sci.

[21] Ramos RM, Burland M, Silva JB, Burman LM, Gelain MS, Debom LM (2019). Photobiomodulation improved the first stages of wound healing process after Abdominoplasty: an experimental, double-blinded, non-randomized clinical trial. Aesthet Plast Surg.

[22] Romanelli M, Piaggesi A, Scapagnini G, Dini V, Janowska A, Iacopi E (2018). Evaluation of fluorescence biomodulation in the real-life management of chronic wounds: the EUREKA trial. J Wound Care.

[23] Traverzim MADS, Makabe S, Silva DFT, Pavani C, Bussadori SK, Fernandes KSP (2018). Effect of led photobiomodulation on analgesia during labor: study protocol for a randomized clinical trial. Medicine (Baltimore).

[24] da-Palma-Cruz M, da Silva RF, Monteiro D, Rehim HMMA, Grabulosa CC, de Oliveira APL (2018). Photobiomodulation modulates the resolution of inflammation during acute lung injury induced by sepsis. Lasers Med Sci.

[25] Langella LG, Casalechi HL, Tomazoni SS, Johnson DS, Albertini R, Pallotta RC (2018). Photobiomodulation therapy (PBMT) on acute pain and inflammation in patients who underwent total hip arthroplasty-a randomized, triple-blind, placebo-controlled clinical trial. Lasers Med Sci.

[26] Baxter GD, Liu L, Petrich S, Gisselman AL, Chapple C, Anders JJ (2017). Low level laser therapy (Photobiomodulation therapy) for breast cancer-related lymphedema: a systematic review. BMC Cancer.

[27] Hamblin MR (2018). Photobiomodulation, photomedicine, and laser surgery: a new leap forward into the light for the 21(st) century. Photomed Laser Surg.

[28] Kuffler DP (2016). Photobiomodulation in promoting wound healing: a review. Regen Med.

[29] Fogacci T, Cattin F, Semprini G, Frisoni G, Fabiocchi L, Samorani D (2018). The use of chromophore gel-assisted blue light phototherapy (Lumiheal) for the treatment of surgical site infections in breast surgery. Breast J.

[30] Nikolis A, Bernstein S, Kinney B, Scuderi N, Rastogi S, Sampalis JS (2016). A randomized, placebo-controlled, single-blinded, split-faced clinical trial evaluating the efficacy and safety of KLOX-001 gel formulation with KLOX light-emitting diode light on facial rejuvenation. Clin Cosmet Investig Dermatol.

[31] Nikolis A, Grimard D, Pesant Y, Scapagnini G, Vézina D (2016). A prospective case series evaluating the safety and efficacy of the Klox BioPhotonic system in venous leg ulcers. Chronic Wound Care Manage Res.

[32] de Freitas LF, Hamblin MR (2016). Proposed mechanisms of photobiomodulation or low-level light therapy. IEEE J Sel Top Quantum Electron.

[33] Sannino M, Lodi G, Dethlefsen MW, Nisticò SP, Cannarozzo G, Nielsen MCE (2018). Fluorescent light energy: treating rosacea subtypes 1, 2, and 3. Clin Case Rep.

[34] Järveläinen H, Puolakkainen P, Pakkanen S, Brown LL, Höök M, Iozzo RV (2006). A role for decorin in cutaneous wound healing and angiogenesis. Wound Repair Regen.

[35] Edge D, Mellergaard M, Dam-Hansen C, Corell DD, Jaworska J, Scapagnini G (2019). Fluorescent light energy: the future for treating inflammatory skin conditions?. J Clin Aesthet Dermatol.

[36] Scapagnini G, Marchegiani A, Rossi G, Zago M, Jowarska J, Wael M, et al. Management of all three phases of wound healing through the induction of fluorescence biomodulation using fluorescence light energy. Proceedings volume 10863 of SPIE (Society of Photo-optical Instrumentation Engineers), Photonic diagnosis and treatment of infections and inflammatory diseases II, 108630W, 7 March 2019, San Francisco, California, US. https://doi.org/101117/12.2508066.

[37] Salvaggio A., Magi GE, Rossi G, Tambella AM, Vullo C, Marchegiani A, et al. Effect of the topical Klox Fluorescence Biomodulation System (Phovia™) on the healing of canine cutaneous incisional wounds. Vet Surg. 2019 (accepted, in press). 10.1111/vsu.13415 32212345

[38] Marchegiani A, Cerquetella M, Laus F, Tambella AM, Palumbo Piccionello A, Ribecco C (2017). The Klox Biophotonic System, an innovative and integrated approach for the treatment of deep pyoderma in dogs: a preliminary report. Vet Dermatol.

[39] Tambella AM, Cerquetella M, Attili AR, Beribè F, Marchegiani A, Palumbo Piccionello A (2017). Klox biophotonic system, a promising innovative approach to canine chronic otitis externa: preliminary report of a randomized controlled clinical trial. Vet Surg.

[40] Nuttal T, Bensignor E (2014). A pilot study to develop an objective clinical score fro canine otitis externa. Vet Dermatol.

[41] Pierezan F, Olivry T, Paps JS, Lawhon SD, Wu J, Steiner JM (2016). The skin microbiome in allergen-induced canine atopic dermatitis. Vet Dermatol.

[42] Moher D, Hopewell S, Schulz KF, Montori V, Gøtzsche PC, Devereaux PJ (2010). CONSORT 2010 explanation and elaboration: updated guidelines for reporting parallel group randomised trials. BMJ..

[43] Giuffrida MA (2014). Type II error and statistical power in reports of small animal clinical trials. J Am Vet Med Assoc.

[44] Di Girolamo N, Meursinge Reynders R (2016). Deficiencies of effectiveness of intervention studies in veterinary medicine: a cross-sectional survey of ten leading veterinary and medical journals. Peer J.

[45] Wareham KJ, Hyde RM, Grindlay D, Brennan ML, Dean RS (2017). Sample size and number of outcome measures of veterinary randomised controlled trials of pharmaceutical interventions funded by different sources, a cross-sectional study. BMC Vet Res.

[46] Antoniou C, Dessinioti C, Sotiriadis D, Kalokasidis K, Kontochristopoulos G, Petridis A (2016). A multicentre randomized, split-face clinical trial evaluating the efficacy and safety of chromophore gel-assisted blue light phototherapy for the treatment of acne. Int J Dermatol.

[47] Nikolis A, Fauverghe S, Vezina D, Scapagnini G (2016). Evaluation of BioPhotonic therapy in a non-healing diabetic foot ulcer: a case report. Diab Foot Can.

[48] Braun SA, Gerber PA (2017). A photoconverter gel-assisted blue light therapy for the treatment of rosacea. Int J Dermatol.

[49] Romanelli M, Piaggesi A, Scapagnini G, Dini V, Janowska A, Iacopi E (2017). EUREKA study - the evaluation of real-life use of a biophotonic system in chronic wound management: an interim analysis. Drug Des Devel Ther.

[50] Mahendran A, Wong XL, Kao S, Sebaratnam DF (2019). Treatment of erlotinib-induced acneiform eruption with chromophore gel-assisted phototherapy. Photodermatol Photoimmunol Photomed.

[51] Lyskova P, Vydrzalova M, Mazurova J (2007). Identification and antimicrobial susceptibility of bacteria and yeasts isolated from healthy dogs and dogs with otitis externa. J Vet Med A Physiol Pathol Clin Med.

[52] Oliveira LC, Leite CA, Brilhante RS, Carvalho CBM (2008). Comparative study of the microbial profile from bilateral canine otitis externa. Can Vet J.

[53] Ngo J, Taminiau B, Fall PA, Daube G, Fontaine J (2018). Ear canal microbiota – a comparison between healthy dogs and atopic dogs without clinical sign of otitis externa. Vet Dermatol.

[54] King SB, Doucette KP, Seewald W (2018). A randomized, controlled, single-blinded, multicenter evaluation of the efficacy and safety of a once weekly two dose otic gel containing florfenicol, terbinafine and betamethasone administered for the treatment of canine otitis externa. BMC Vet Res.

[55] Hill PB, Lau P, Rybnicek J (2007). Development of an owner-asisted scale to measure the severity of pruritus in dogs. Vet Dermatol.

[56] Rybníček J, Lau-Gillard PJ, Harvey R, Hill PB (2009). Further validation of a pruritus severity scale for use in dogs. Vet Dermatol.

[57] Hill P, Rybníček J, Lau-Gillard P (2010). Correlation between pruritus score and grossly visible erythema in dogs. Vet Dermatol.

[58] Buback JL, Boothe HW, Carroll GL, Green RW (1996). Comparison of three methods for relief of pain after ear canal ablation in dogs. Vet Surg.

[59] Grono LR (1970). Studies of the microclimate of the external auditory canal in the dog. I Aural temperature. Res Vet Sci.

[60] Cole LK (2010). Anatomy and physiology of the canine ear. Vet Dermatol.

[61] Mittal A, Kumar S (2014). Role of pH of external auditory canal in acute otitis externa. Indian J Otolaryngol Head Neck Surg.

[62] Ferguson AW, Scott JA, McGavigan J, Elton RA, McLean J, Schmidt U, Kelkar R, Dhillon B (2003). Comparison of 5% povidone-iodine solution against 1% povidone-iodine solution in preoperative cataract surgery antisepsis: a prospective randomized double blind study. Br J Ophthalmol.

